# Motifs in the permeation pathway of connexin channels mediate voltage and *Ca*^2+^ sensing

**DOI:** 10.3389/fphys.2014.00113

**Published:** 2014-03-31

**Authors:** Andrew L. Harris, Jorge E. Contreras

**Affiliations:** Department of Pharmacology and Physiology, New Jersey Medical School, Rutgers UniversityNewark, NJ, USA

**Keywords:** connexin, hemichannels, voltage gating, gap junction channels, permeation, *Ca*^2+^ regulation

## Abstract

Connexin channels mediate electrical coupling, intercellular molecular signaling, and extracellular release of signaling molecules. Connexin proteins assemble intracellularly as hexamers to form plasma membrane hemichannels. The docking of two hemichannels in apposed cells forms a gap junction channel that allows direct electrical and selective cytoplasmic communication between adjacent cells. Hemichannels and junctional channels are gated by voltage, but extracellular *Ca*^2+^ also gates unpaired plasma membrane hemichannels. Unlike other ion channels, connexin channels do not contain discrete voltage- or *Ca*^2+^–sensing modules linked to a separate pore-forming module. All studies to date indicate that voltage and *Ca*^2+^ sensing are predominantly mediated by motifs that lie within or are exposed to the pore lumen. The sensors appear to be integral components of the gates, imposing an obligatory structural linkage between sensing and gating not commonly present in other ion channels, in which the sensors are semi-independent domains distinct from the pore. Because of this, the structural and electrostatic features that define connexin channel gating also define pore permeability properties, and *vice versa*; analysis/mutagenesis of gating and of permeability properties are linked. This offers unique challenges and opportunities for elucidating mechanisms of ligand and voltage-driven gating.

## Connexin channels: an overview

Connexin proteins form channels in plasma membrane and between cells that are permeable to atomic ions and small molecules. The connexin channels between cells (gap junction channels, GJCs) are formed by extracellular docking of two hexameric hemichannels. GJCs allow propagation of electrical and molecular signaling among neighboring cells (Bennett et al., 1991). Unpaired hemichannels play an autocrine/paracrine role by releasing transmitters, such as glutamate and ATP, into the extracellular environment (Bennett et al., 2003; Wang et al., 2013).

The gating and permeability properties of these channels are highly specific and tightly regulated; mutations that alter these properties cause human pathologies (cf., Zoidl and Dermietzel, 2010; Pfenniger et al., 2011). Contrary to early notions, connexin channels are not just “simple pores” (i.e., always open and nonspecifically permeable to molecules below a size cut-off). They fluctuate among open and closed states in a regulated manner, they are sensitive to membrane and junctional voltages and to divalent ions, and their permeability properties can be surprisingly selective among molecules with similar size (Harris, 2001). They differ substantially from most other voltage and *Ca*^2+^ gated channels in two key ways (*in addition* to forming junctional channels):

1. The identity of the channel is not defined by a specific ionic selectivity. That is, the pore of a *K^+^* channel is optimized to be highly selective for *K^+^* and to select against other ions, whereas the pore of connexin channels is not constrained to be selective for only one particular ion or molecule. The different connexin channels have pores with widely divergent yet specific properties, as reflected in unitary conductances ranging from 17 to over 300 pS (for GJCs) and greatly different molecular permeabilities (Harris, 2001, 2007). Perhaps surprisingly, the unitary conductances (a function of permeability to atomic ions, *K^+^* and *Cl*^−^) do not correlate with apparent limiting pore diameter (e.g., some connexin channels with high conductance are highly size-restrictive, and *vice versa*), and neither property correlates simply with permeability to specific molecules (e.g., fluorescent tracers, metabolites such as ATP, cGMP, etc.). Indeed, it is clear that size restriction and charge selectivity alone cannot account for the molecular selectivity data. The existing data suggest that the pores are optimized for specific molecular permeability characteristics; it is a challenge and biomedically important to determine precisely what those are and how disease-causing mutations alter them.
2. The primary sensors that control gating by voltage (for GJCs and hemichannels) and extracellular *Ca*^2+^ (for hemichannels) are likely within or exposed to the pore. Connexin channels do not have the modular structure commonly seen in other channels in which, for example, a voltage sensing module is structurally distinct from the pore, or a large extracellular or cytoplasmic domain mediates ligand binding. The voltage and *Ca*^2+^ sensors in connexin channels seem to be within the aqueous pore. Furthermore, it appears likely that the sensors and the physical “gates” they operate are the same or overlapping structures. A direct consequence is that the structures that mediate voltage and extracellular *Ca*^2+^ sensing (and mutations in those structures) can affect the permeability properties of the pore, and *vice versa*. Thus, unlike most voltage-gated channels, in connexins the structural and electrostatic parameters that determine channel conductance properties and gating charge movement are closely linked and interact.

The properties of connexin channels are defined by their specific composition. There are 21 human connexin isoforms (Willecke et al., 2002). Almost all cellular types, with a few exceptions such as erythrocytes, express one or more isoform; in fact, most cells express more than one isoform, and when two compatible isoforms are expressed in the same cell, the hemichannels are heteromeric. Heteromeric connexin channels can have properties not simply predicted from those of the corresponding homomeric channels. For a given tissue, the expression of specific connexin isoform(s), their relative abundance(s), their ability to form heteromeric hemichannels and the specificity of hemichannel-hemichannel docking to form GJCs define the unique and proper function of the channels in that tissue, and are key to normal physiology. The importance of such specificity is demonstrated by studies in which genetic replacement of one connexin by another (knock-in) typically fails to recapitulate wild-type tissue/organ function (Plum et al., 2000; Martinez-Wittinghan et al., 2003; Frank et al., 2010; Dicke et al., 2011).

As expected from the broad tissue distribution and the role of connexin channels in intercellular signaling, mutations in connexin genes are associated with a significant number of human diseases including deafness, cataracts, cardiac and developmental defects, skin and neurological disorders (Abrams and Scherer, 2012; Delmar and Makita, 2012; Xu and Nicholson, 2013). More than 100 pathology-causing mutations have been described in the gene for one of the smallest human connexins (connexin26; hCx26; gene *GJB2*). Most of these mutations are functional nulls, which are responsible for ~50% of inherited childhood nonsyndromic recessive hearing loss. However, in those cases where the mutated gene yields functional Cx26 channels, the deafness is predominantly associated with congenital skin disorders, such as Vohwinkel's syndrome, Keratitis-ichthyosis-deafness syndrome (KID), and palmoplantar keratodermas (Lee and White, 2009; Martinez et al., 2009; Xu and Nicholson, 2013).

For functioning mutants of hCx26 and other connexins that cause human pathology, the resulting channels generally have altered gating of hemichannels and GJCs and/or evidence of altered molecular permeability (Lee and White, 2009; Martinez et al., 2009). Dysfunction of the signaling communication mediated by connexin channels can also become evident only in the context of other nongenetic pathological conditions, such as ischemia, trauma/inflammation, and neuropathic pain, in which unbalanced or altered connexin expression or function potentiates tissue dysfunction and damage (De Maio et al., 2002; Contreras et al., 2004; Bennett et al., 2012; Chen et al., 2012).

The section immediately below summarizes the structural context for discussion of voltage and extracellular *Ca*^2+^ sensing by connexin channels, focusing on hCx26, the only connexin for which there is a reasonably high-resolution structure. The sections that follow present detailed information on these processes. This review will not address structural issues of hemichannel-hemichannel docking (Bai and Wang, 2014), nor the mechanisms of connexin channel regulation by pH_*i*_ (Morley et al., 1997) or intracellular *Ca*^2+^ (for which the targets seem to be cytosolic and outside the pore; the latter likely mediated by interaction with calmodulin) (Zou et al., 2014). A recent comprehensive review of connexin channel function, cell biology, and biomedical involvement may be found in Nielsen et al. (2012).

## Structural context of connexin channel gating by extracellular *Ca*^2+^ and by voltage

Under normal conditions, undocked hemichannels in plasma membrane are crucially maintained in a predominantly closed state by physiological extracellular *Ca*^2+^ (~1.8 mM). This is obviously important for cellular survival, since due to the large size and modest charge selectivity of the aqueous pore, exacerbated opening of unapposed connexin hemichannels at the plasma membrane leads to loss of electrochemical gradients and of small cytoplasmic metabolites, causing cell death (Saez et al., 2010; Fasciani et al., 2013). A host of studies demonstrate that even modest reduction of extracellular *Ca*^2+^ below normal levels can result in substantially increased hemichannel open probability (Ebihara and Steiner, 1993). The opening of hemichannels is also influenced by membrane voltage, with depolarization favoring channel opening (Saez et al., 2005; Gonzalez et al., 2007; Fasciani et al., 2013). The potential interaction of these two factors in controlling hemichannel open probability is a matter of active investigation.

**Figure 1:**
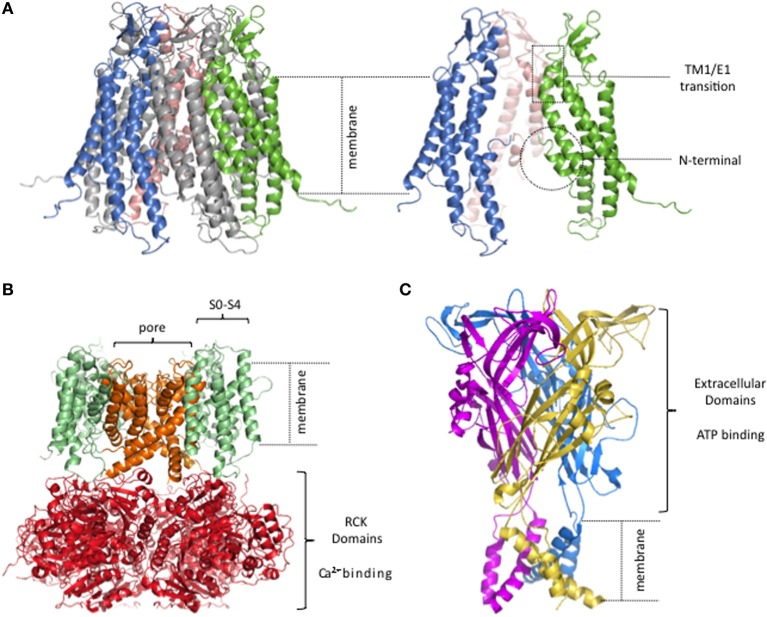
**Lack of semi-independent structural modules in connexin hemichannels**. **(A)** Human Cx26 hemichannel derived from crystal structure of Maeda and colleagues (PDB code. 2ZW3; Maeda et al., 2009). The portions that were unresolved in the crystal structure were added and the completed structure equilibrated by molecular dynamics in explicit membrane and water. structure. (Kwon et al., 2011). Right, side view of 3 connexin subunits to better visualize the pore of the hemichannel. The TM1/E1 region and the NT domains are indicated. **(B)** Homology model of a BK channel, modified from (Contreras et al., 2013). Four subunits form the channel. The pore, and the RCK and voltage sensor domains are highlighted in different colors. **(C)** Crystal structure of the P2X4 channel (PDB code: 3H9V; Kawate et al., 2009). Each subunit is depicted in a different color.

Despite long-standing knowledge of these gating modulators, we still have only a modest understanding of the motifs that regulate opening and closing of connexin channels and the corresponding conformational dynamics. A major advance, in this respect, is the solution of the hCx26 gap junction channel structure at 3.5 Å by X-ray crystallography (Figure 1A) (Maeda et al., 2009). Although this resolution can show only the basic contours of the protein chain, the refinement of the structure is consistent with electrophysiological, mutagenesis and biochemical data that inferred structural topology and certain structure-function relations. The connexin channel topology consists of four membrane-spanning segments (TM1-TM4), with the C-terminal (CT) and N-terminal (NT) domains toward the intracellular side. Three loops connect the transmembrane segments, one cytoplasmic (CL), and two extracellular (E1 and E2). The solved structure is consistent with the ion permeation pathway being lined—starting at the cytoplasmic entrance—by the first half of the NT folded into the pore lumen as an α-helix, stabilized by interactions with TM1, then by TM2, followed by the first half of E1 at the extracellular end of the hemichannel. The narrowest part of the pore diameter in the crystal structure is estimated at ~14 Å and is located at the constriction formed by the NT residue about halfway through the pore. The folded structure of the NT was supported by prior peptide NMR studies of this domain (Purnick et al., 2000a) and has been bolstered by subsequent work (Kalmatsky et al., 2009, 2012). Given the absence of an obvious occlusion of the lumen by any part of the solved structure, it is presumed to correspond to an open state of the channel. It is important to note that several parts of the protein were unresolved in the structure, including almost all of the CL, the short CT and the N-terminal methionine [shown to be present and acetylated in this protein by MS/MS (Locke et al., 2009)]. The CL and the CT have been shown to be modulators of gating in connexins, though they are unlikely to be directly involved in the gating mechanisms discussed here.

X-ray crystal structures solved for other ligand- and voltage-gated channels show a different sort of organization, an obvious feature of which is well-defined structural modules in which the ligand and voltage sensors are structures semi-independent from the pore. As an example, Figure 1 shows a typical structural model for a *Ca*^2+^ and voltage activated potassium channel (BK channel; Figure 1B) and an ATP-gated channel (Figure 1C).

The BK channel has a voltage sensor that resides within the plasma membrane, formed by TM1-TM4 [also named S1–S4 (Diaz et al., 1998)] while the pore domain is formed by a different part of the protein, TM5-TM6. The *Ca*^2+^ binding pocket is located in the large C-terminal intracellular region where eight Regulator of Conductance for *K^+^* (RCK) domains form a ring (Wu et al., 2010). The connexin structure has no obvious distinct structural modules within the membrane, one for voltage sensing and another for ion conduction, nor an extramembranous *Ca*^2+^ binding domain connected to the pore.

The ATP-gated channel senses extracellular ATP via a large extracellular region, harboring three potential ATP-binding pockets, disulfide-rich motifs, and vestibules that form a void in the middle of three subunits (Kawate et al., 2009; Hattori and Gouaux, 2012). Sensing of extracellular *Ca*^2+^ by such a large extracellular ligand binding domain would not be possible for connexin channels since it would interfere with the extracellular docking of two hemichannels from adjacent cells required to form a GJC.

In connexin channels, the NT and the connecting region of TM1/E1 have been implicated as gating transducers of voltage and extracellular *Ca*^2+^ sensing, respectively, as well as possible constituents of the gates (Bargiello et al., 2012; Lopez et al., 2013a). That they also form part of the permeation pathway (cf., Trexler et al., 2000; Oh et al., 2008), suggests a structural organization that is not common in other channels.

The relation between the published connexin crystal structure and the permeability properties were recently assessed by Grand Canonical Monte Carlo Brownian dynamics (GCMC/BD) (Kwon et al., 2011). To perform these calculations meaningfully, the missing regions and residues of the channel that were unresolved in the crystal structure were added and the structure minimized in explicit membrane and solvent. GCMC/BD simulations showed that the completed structure was nearly nonconductive and almost perfectly anion selective, both properties largely due to the presence of the N-terminal methionine, which severely narrowed the pore. All-atom molecular dynamics (MD) simulations significantly relaxed the structure, resulting in more appropriate unitary conductance and less anion selectivity (Kwon et al., 2011). The completed and MD-equilibrated structure appears to more closely represent the open Cx26 hemichannel structure than does the crystal structure and co- and posttranslational modifications of the protein are proposed to play an important physiological role by defining the conductance and ion selectivity of the channels. Further experimentation is needed to confirm these notions.

## Gating by extracellular *Ca*^2+^

Reduction of extracellular *Ca*^2+^ increases opening of most, if not all, unapposed connexin hemichannels (i.e., Cx26, Cx32, Cx43, Cx46, and Cx50) in plasma membrane (Paul et al., 1991; Zampighi et al., 1999; Valiunas and Weingart, 2000; Beahm and Hall, 2002; Contreras et al., 2003; Ebihara et al., 2003; Gomez-Hernandez et al., 2003; Ripps et al., 2004; Lopez et al., 2013a). Extracellular divalent cation concentrations are considered the primary physiological mechanism to protect cells from the adverse effects of substantial hemichannel opening. In most connexins, at physiological concentrations, extracellular *Ca*^2+^, rather than *Mg*^2+^, is the more effective divalent ion in keeping connexin hemichannels closed (Ebihara et al., 2003; Lopez et al., 2013b). Estimates for the apparent *Ca*^2+^ affinity for hemichannel inhibition, based on electrophysiological studies, for several connexins heterologously expressed in oocytes range from 0.15 to 1.3 mM. Direct methods for measuring *Ca*^2+^ binding to hemichannels, such as isothermal calorimetry, have not been applied to date, perhaps due to the potential for precipitation of the high concentration of connexin protein required, in the presence of millimolar *Ca*^2+^.

Several other divalent and trivalent cations have been shown to inhibit connexin hemichannel activity. The experimentally determined rank order of potency varies among different connexins, but in general is: *La*^3+^ ≈ *Gd*^3+^ > *Zn*^2+^ > *Cd*^2+^ > *Co*^2+^ > *Ca*^2+^ > *Mg*^2+^ > *Ba*^2+^ (Eskandari et al., 2002; Ebihara et al., 2003; Verselis and Srinivas, 2008; Sun et al., 2009; Fasciani et al., 2013). However, these results may be complicated by the fact that some metal ions, including *Zn*^2+^ and *Cd*^2+^, are also likely to interact with amino acid residues such as cysteine and histidine at other sites to affect gating in ways that are unrelated to physiological regulation by *Ca*^2+^ (Chappell et al., 2004).

The detailed mechanisms by which changes in extracellular *Ca*^2+^ affect hemichannel open probability remain unknown. Low-resolution structural methods have long shown conformational changes of connexin channels induced by *Ca*^2+^ (Unwin and Ennis, 1983; Muller et al., 2002). Electron microscopic analysis of isolated rat liver GJCs at 2.5 nm resolution showed significant differences in the pore size between the *Ca*^2+^ and *Ca*^2+^-free structures, leading the authors to suggest that *Ca*^2+^ causes a rotation of the rigid subunits that closes the pore in manner analogous to the closing of a camera iris. More recently, atomic force microscopy of isolated mouse Cx26 hemichannels showed an increase in the pore diameter from 2.5 to 5 nm when *Ca*^2+^ is removed from the solution, suggesting that *Ca*^2+^ stabilizes a closed conformation of the channels (Muller et al., 2002). Similar large rearrangements in response to changes in *Ca*^2+^ at the entrances of the pore in Cx40 and Cx43 have been reported using the same methodology (Thimm et al., 2005; Allen et al., 2011). However, in these types of preparations *Ca*^2+^ can access both the extracellular and cytoplasmic domains of the channels, so the molecular target of *Ca*^2+^ binding is not revealed. To date, identification of the extracellular site(s) of *Ca*^2+^ binding in connexin hemichannels has been hindered by the lack of published high-resolution structural data in the presence of *Ca*^2+^.

High and low affinity *Ca*^2+^ binding sites are found in several other proteins. Most of these sites are typically in globular or “lobe” domains, in which *Ca*^2+^ ions are coordinated almost entirely by the oxygen atoms of negatively charged residues (Asp or Glu). The existing hCx26 crystal structure, which was obtained in the absence of *Ca*^2+^ and presumably represents a channel with open gates, does not reveal an obvious *Ca*^2+^ binding site. This could mean that the formation of the *Ca*^2+^ binding site is structurally linked to formation of a closed state.

The precise location of the physical gate that opens and closes in response to changes in extracellular *Ca*^2+^ concentration is unknown. One electrophysiological study using cysteine mutagenesis at a position within the pore (Leu-35) in Cx46, along with chemical modification, showed maleimide accessibility from the intracellular, but not extracellular side of the pore, in the presence of millimolar *Ca*^2+^. Since Leu-35 is located at the middle of the pore, it was suggested that the gate is located toward the extracellular side of the pore (Pfahnl and Dahl, 1998). This an intriguing observation, however, to firmly establish the position of the *Ca*^2+^ regulated gate the accessibility pattern of multiple residues along the entire pore length and the rates of modifications, in the presence and absence of *Ca*^2+^, are needed.

Barrio and colleagues showed that mutations of Asp-169 and Asp-178 at the extracellular vestibule significantly reduce the ability of extracellular *Ca*^2+^ to close Cx32 hemichannels (Gomez-Hernandez et al., 2003). It was proposed that residue Asp-169 of one subunit and Asp-178 of an adjacent subunit must be arranged precisely to allow effective interactions with *Ca*^2+^. However, many other connexins do not have a negatively charged or even a polar residue at the equivalent site of Asp-169, yet are strongly regulated by extracellular *Ca*^2+^. This suggests that these findings do not stand for a general mechanism of *Ca*^2+^ control of hemichannels, but may be of specific importance for Cx32. Also, different connexin channels may have multiple regulatory binding sites.

In human Cx37, the effect of extracellular *Ca*^2+^ was found to be voltage-dependent, which also suggests that *Ca*^2+^ binds within the ion permeation pathway (Puljung et al., 2004). It was proposed that *Ca*^2+^ acts as an intra-pore gating particle that forms part of the gate. In an attempt to elucidate the *Ca*^2+^ gating mechanism, another study suggests that “loop gating,” in Cx46 hemichannels, corresponds to intrinsic conformational changes of the connexin pore, and that extracellular divalent ions are modulators of these voltage-dependent structural rearrangements (Verselis and Srinivas, 2008). The unidentified intrinsic gate can fluctuate between open and closed and is voltage-sensitive, but is dramatically stabilized when *Ca*^2+^ binds to the closed conformation suggesting that *Ca*^2+^ acts an allosteric inhibitor of the channels (Verselis and Srinivas, 2008).

Recently, we found that extracellular *Ca*^2+^ destabilizes the open state of hemichannels, at least in hCx26 and hCx30, by disrupting salt bridge interactions located at the extracellular entrance of the pore. The open state destabilization facilitates hemichannel closure (Lopez et al., 2013a,b). Human Cx26 mutations at position Asp-50 (Asn/Tyr-50) that produce diseases reveal that this residue is a key regulator of *Ca*^2+^ interactions by forming a salt bridge with position Lys-61 in an adjacent subunit that stabilizes the open state, and that this interaction is sensitive to changes in extracellular *Ca*^2+^ concentration. Similar studies indicate that this salt bridge can also interact with Gln-48 to further stabilize the open state (Lopez et al., 2013b; Sanchez et al., 2013). Mutagenesis studies suggest that other charged residues form an electrostatic network at the entrance of the pore that can play a role in the gating dynamics of pore (Tong et al., 2013). The hCx26 crystal structure and molecular dynamics studies based on it support the idea of an electrostatic network located at the extracellular entrance of the connexin pore that is highly conserved among different connexins (Kwon et al., 2012). It is likely that extracellular *Ca*^2+^ directly or indirectly destabilizes these networks to favor a closed conformation. It should be noted that these studies cannot identify the specific amino acid residues that coordinate the bound *Ca*^2+^. So far, all one can say is that they are involved in *Ca*^2+^ sensitivity. However, it is very likely that both *Ca*^2+^ binding and the conformational changes that follow to occlude the pore occur in this region. It is possible that the *Ca*^2+^ binding site(s) is formed upon sequential pore rearrangements occurring from the open to the closed channel conformation.

Since all evidence points to the regulatory *Ca^2^*^+^ being coordinated by residues in different connexin subunits, a potential molecular mechanism for divalent regulation is suggested by recent work on the CorA *Mg*^2+^ transporter. CorA activity is inhibited by *Mg*^2+^ binding at a modulatory site formed by Asp residues from different subunits. In CorA, the effect of *Mg*^2+^ binding is to reduce electrostatic repulsion between the subunits, which allows formation of stabilizing salt-bridge interactions that stabilize an open permeation/transport pathway. While the regulatory *Mg*^2+^ site in CorA is not exposed to the pore, and effects on salt-bridge formation and pore opening are allosteric rather than local to the *Mg*^2+^ binding site, modulation of intersubunit contacts by a divalent may be relevant to *Ca^2^*^+^ regulation of connexin channels (Lunin et al., 2006; Pfoh et al., 2012; Payandeh et al., 2013).

## Voltage gating

GJCs and unpaired hemichannels display two forms of intrinsic voltage dependent gating, termed V_*j*_ and “loop” gating (Trexler et al., 1996; Gonzalez et al., 2007; Bargiello et al., 2012). In GJCs, these gating mechanisms sense the potential difference between the cytoplasms of adjacent cells, referred to as the *transjunctional voltage*, while in unpaired hemichannels they sense the *transmembrane voltage*. However, GJCs are almost insensitive to the *transmembrane voltage*, implying that the voltage that is sensed is within the pore. This idea, along with the idea that each component hemichannel contains discrete voltage sensors, was strongly supported by early studies (Harris et al., 1981; Spray et al., 1981) and confirmed quantitatively by later work (cf., Bukauskas et al., 2001; Paulauskas et al., 2009). While it is conceivable that the docking of two hemichannels could create a unified transjunctional voltage sensor that spans both membranes and is separate from the pore, there is no structural or electrophysiological evidence for this, and there is abundant evidence for voltage sensing within the pore in both hemichannels and GJCs. Voltage sensing within the pore allows conservation of V_*j*_ and “loop” gating sensitivities in both GJCs and hemichannels.

The voltage sensitivities of these two gating mechanisms can be dissociated by mutagenesis (Verselis et al., 1994; Purnick et al., 2000b; Oh et al., 2004) however the exact and complete molecular identities of voltage sensors are unknown. Empirically, V_*j*_ and “loop” gating are defined by the characteristics described below:

1. V_j_ gating governs rapid transitions to and from a substate. Functional studies show that mutations in the NT alter the voltage polarity of gating to substates, suggesting that it is a constituent of the voltage sensor that mediates V_j_ gating voltage sensitivity (Figure 1A).
2. “Loop” gating mediates slow transitions to and from fully closed state(s). It is associated with conformational changes at and just beyond the TM1/E1 transition at the extracellular part of the pore (Figure 1A). As noted above, this region is also implicated in the hemichannel *Ca*^2+^ sensitivity and has been suggested to form part of the gate.

The voltage-dependent substate of V_j_ gating is intimately linked to the properties of the NT. Functional studies show that mutations in the NT, which was identified by biochemical studies to be cytoplasmic (Hertzberg et al., 1988; Milks et al., 1988; Falk et al., 1994), alter the polarity of the voltage controlling gating to and from substates. These studies focus mainly on Cx26 and Cx32 hemichannels, where the presence of a charged residue (positive or negative) in the second residue of the NT can switch the polarity of the voltage gating (Rubin et al., 1992a,b; Verselis et al., 1994; Purnick et al., 2000b; Oh et al., 2004). The results essentially establish the NT as a key constituent of the voltage sensor. Since the mutated residues near the N-terminus cannot sense voltage if they are outside the membrane field, it was suggested that the NT folds into the pore lumen. Such a bent or folded structure of this domain is supported by peptide NMR studies (Purnick et al., 2000a; Kalmatsky et al., 2009, 2012). The NT domain may be quite flexible, since antibody-binding and enzymatic cleavage show that it is accessible from the aqueous phase, yet the structural studies show it within the pore.

The crystal structure of hCx26 GJCs shows precisely this structure, with much of the NT located within the ion permeation pathway and so in a position to sense changes of the *transmembrane potential* (and *transjunctional voltage* for GJCs). The location of much of the NT region within the pore was initially proposed by studies using a 3D map derived from 2D crystals analyzed by cryoelectron microscopy (resolution 10 Å in the membrane plane and 14.1 Å normal to the membrane plane) of hCx26 with a point mutant in TM1 (Oshima et al., 2007). This map shows a prominent density in the pore of each hemichannel, forming a “plug,” and it was suggested that it was composed of the NT folded into the lumen of the pore from the cytoplasmic side. Further studies supported this idea (Oshima et al., 2011). The “plug” structure, along with the higher-resolution structure of the wild-type hCx26, led to the inference that voltage-driven movement of the NT in the pore could lead to two structures, one in which the NT is folded against the inner wall, allowing a patent pore, and another in which the stabilizing interactions with the pore wall were lost, with the NTs collapsing into a plug that occludes the lumen. Given abundant data showing that the charge near the end of the N-terminus alters the polarity of voltage sensitivity, this mechanism was proposed to mediate a form of voltage gating (Maeda et al., 2009). It also brings into high relief the interdependence of voltage gating and pore properties.

Interestingly, cytosolic domain interactions, specifically, between the CT and the CL domains, seem to critically affect voltage dependent sub-states in Cx40 and Cx43 (Anumonwo et al., 2001; Moreno et al., 2002; Seki et al., 2004; Shibayama et al., 2006). Chimeric proteins in which the CLs and CTs of Cx26 and Cx30 are swapped have altered gating and voltage sensitivity, even though these domains are cytosolic (Manthey et al., 2001). Simple modifications such as truncation or linking of green fluorescent protein to the CT of Cx43 can eliminate occupancy of observable sub-states sensitive to voltage (Bukauskas et al., 2001; Contreras et al., 2003). Addition of a small epitope to the CT of Cx43 can alter voltage gating (Desplantez et al., 2011). However, these CT effects may be specific to certain connexin isoforms, since truncation of a Cx32-based channel does not alter voltage gating (Kwon et al., 2013a).

Another important property of voltage dependent sub-states described in Cx43 GJCs is the change in permeability with respect to the full channel conductance. Bukauskas and colleagues show that monovalent positively and negatively charged molecules that are permeable to the full channel conductance do not permeate voltage dependent sub-states (Bukauskas et al., 2002). It is unknown whether these changes in the permeability properties correlate with structural rearrangements of the NT inside the pore. A number of mutations at the NT have been shown to affect selectivity of small molecules, voltage dependence and unitary conductance, expected if the NT is folded within the pore (Xin et al., 2010; Beyer et al., 2012; Schlingmann et al., 2012; Xin and Bai, 2013).

Recently, chimera studies swapping the NT of Cx46 and Cx50 showed significant changes in unitary conductance (Kronengold et al., 2012). In addition to the NT, substitution of the E1/TM2 domains of Cx46 into a Cx50 background converted the weak “loop” gating of Cx50 into a stronger form resembling that of Cx46 hemichannels. Similar results have been observed in chimeras between the chick connexin homologs, Cx45.6 and Cx56 (Tong et al., 2004; Tong and Ebihara, 2006) and chimeras between Cx32 and Cx37 (Hu et al., 2006). The most notable fact is that substitution of NT alone also influences “loop” gating, indicating that there is an intimate linkage between the NT and other parts of protein, perhaps via TM1, to the initial segment of E1 (Kronengold et al., 2012).

Consistently, conformational dynamics in response to changes in *transmembrane voltage* have been reported at the TM1/E1 region, and more recently at the intracellular side, in hemichannels (Tang et al., 2009; Verselis et al., 2009; Kwon et al., 2013b). Chemical modification using thiol reagents, metal ion binding, and cysteine scanning mutagenesis has been applied to investigate pore-lining residues and the nature of the connexin interactions that result in a closed “loop” gate. A widening of the pore is observed between residues 40 and 50, in the TM1-E1 region, in Cx50 and chimeric Cx32*43E1 hemichannels (in which the E1 of Cx43 replaces that of Cx32) during depolarizing voltage pulses. Unfortunately, the role of this region as directly forming an impermeable pore barrier has not yet been conclusively demonstrated using these methodologies; they are undoubtedly intimately involved in the process. To date, technical limitations in measuring rates of thiol modification and its correlation with open channel probability prevent an interpretation that rigorously and unequivocally identifies the gate. For example, in excised patches, it is not possible to adequately control the open probability of connexin hemichannels due to rapid run down. In addition, for macroscopic hemichannel currents chemical modification shows variability and reversibility of MTS and metal ion binding (likely due to channel permeability to cellular reducing agents; (Tong et al., 2014) that complicates precise determination of a rate.

In summary, the evidence suggests that voltage sensing occurs within the ion permeation pathway of the connexin channels. This notion is not entirely unique to connexins. It is now well established that voltage sensing of a variety of *K^+^* channels occurs at the pore region most likely at the selectivity filter, which also serve as the gate (Cordero-Morales et al., 2006; McCoy and Nimigean, 2012; Posson et al., 2013). These *K^+^* channels lack the canonical voltage sensor domain found in Kv channels, which is formed by TM1 to TM4. The natural coupling between the voltage sensor and channel gating occurring within the ion permeation pathway may, by default, affect channel selectivity. Mutagenesis studies in Kir6.2 channels support this idea, in which mutations located at the permeation pathway affect voltage sensitivity (Kurata et al., 2010) as well as channel selectivity. A different type of example is provided by voltage-gated proton channels (Hv channels) in which a charge-bearing arginine of a classical voltage-sensing S4 domain interacts with another residue to form/break the “selectivity” filter/gate for proton flux as it moves in a voltage field (Decoursey, 2013; Gonzalez et al., 2013; Chamberlin et al., 2014; Li et al., 2014). Exploration of how voltage sensing, gating, and selectivity can operate as an integrated ensemble will be challenging and informative.

## Future steps

There are several outstanding issues regarding the mechanisms of *Ca*^2+^ and voltage gating in connexin channels that remain to be solved. These include: (a) The molecular identity of the *Ca*^2+^ and voltage sensors; (b) The structural linkage between the sensors and the as yet unidentified gate(s); (c) Whether the *Ca*^2+^- and voltage-triggered gating structural rearrangements are identical or if these present variations that lead to different properties of the pore. The existing structural maps do not provide sufficient information to direct functional studies to address these issues.

One of the most challenging aspects is resolution of the gating sensitivities and conformational changes that take place at the TM1/E1 transition. As indicated above, this pore-exposed region is known to undergo changes during both extracellular *Ca*^2+^ and (“loop”) voltage sensing/gating. Most studies suggest that gating involving this region has an intrinsic voltage sensitivity and an intrinsic *Ca*^2+^ sensitivity, but the structural and thermodynamic interactions among them with the associated changes in gating status remain to be elucidated. Both *Ca*^2+^ binding and voltage sensitivity are likely to be state dependent. Given the highly localized nature of the key region, it may be difficult if not unrealistic to fully tease apart these processes; they may be structurally highly integrated. An understanding of gating at this region may require close synergy of computational and experimental approaches.

A related issue is the how changes in the NT, CL or CT (e.g., mutation, deletions, truncations), which are on the other side of the membrane from the TM1/E1 region, are able to affect regulation of the channels by *Ca*^2+^ and “loop” gating. These effects could most simply derive from state-dependence of the sensitivities mediated by TM1/E1, but may also be mediated by alterations in the distribution of charge, voltage profile, or ion occupancy within the pore. For example, several disease-causing mutations in the TM1/E1 region result in plasma membrane hemichannels that are aberrantly open and empirically less sensitive to extracellular *Ca*^2+^, but the same general channel phenotype is caused by certain mutations in the cytoplasmic region of the NT. How these latter mutations affect binding of extracellular *Ca*^2+^ (e.g., by affecting the microscopic binding affinity itself or the state-dependence of the binding site) or the conformational/gating changes that follow extracellular *Ca*^2+^ binding is unknown.

Understanding of the gating mechanisms of connexin channels is less advanced than for most other ion channels. The experimental strategies applied so far to connexin channel structure-function appear relatively simple compared with the sophisticated strategies now often applied to other ion channels. The primary reason is that connexin channels, whether junctional or hemichannels, present substantial, and unique technical difficulties for quantitative biophysical studies. For instance, mutagenesis studies aiming to reveal the motifs that control a particular gating reaction must consider that the mutations are likely to affect other properties that are also defined by the pore (e.g., voltage sensing by loop and V_j_ mechanisms, divalent ion coordination, substate occupancy, unitary conductance, molecular selectivity), in addition to the expected potential allosteric effects that one must consider for mutagenesis studies of any channel.

On the other hand, the overlapping nature of the determinants of connexin channel properties can provide additional readouts about the effects of the mutations (e.g., changes in unitary conductance, or charge selectivity) that may aid in understanding the structural changes. Perhaps more than for other channels, understanding how connexin channels work requires an integrated, rather than modular, analytical strategy. Beyond the profound biomedical significance of understanding the regulatory mechanisms of connexin channels, and the insights about membrane protein dynamics derived from them, further studies aiming to reveal molecular gating mechanisms should also serve to inform new pharmacological strategies to treat connexin mutation-causing diseases.

### Conflict of interest statement

The authors declare that the research was conducted in the absence of any commercial or financial relationships that could be construed as a potential conflict of interest.
